# Proximal femoral morphometry and its association with hip fracture patterns: a systematic review with meta-analysis

**DOI:** 10.1007/s00276-026-03898-8

**Published:** 2026-05-22

**Authors:** Dimitrios Kotzias, Vasileios Giovanoulis, Christos Koutserimpas, George Triantafyllou, Enejd Veizi, Nikolaos-Achilleas Arkoudis, George Tsakotos, Theodore Troupis, Maria Piagkou

**Affiliations:** 1 Department of Anatomy, School of Medicine, National and Kapodistrian University of Athens, 75 Mikras Asias str., Goudi, 11527, Athens, Greece; 2 Department of Orthopaedics and Traumatology, ‘251’ Hellenic Air Force General Hospital of Athens, Athens, Greece; 3 Lyon Ortho Clinic, Clinique de la Sauvegarde, Lyon, France; 4 School of Health Rehabilitation Sciences, University of Patras, Patras, Greece; 5 2nd Department of Orthopaedic Surgery, ‘Hygeia’ General Hospital of Athens, Athens, Greece; 6 Department of Orthopaedics and Traumatology, Ankara Yildirim Beyazit University, Ankara, Turkey; 7 Research Unit of Radiology and Medical Imaging, National and Kapodistrian University of Athens, Athens, Greece; 8 Second Department of Radiology, General University Hospital “Attikon”, National and Kapodistrian University of Athens, Athens, Greece

**Keywords:** Proximal femoral morphometry, Hip fractures, Hip fracture patterns, Radiographic geometry, Elderly patients

## Abstract

**Purposes:**

Proximal femoral fractures (PFFs) represent a major cause of morbidity and mortality in the elderly population. Beyond bone mineral density, proximal femoral (PF) and hip joint (HJ) morphometry have been implicated in fracture susceptibility and fracture pattern. However, the relative contribution of individual geometric parameters remains incompletely defined. The purpose of this study was to systematically evaluate the association between PF and HJ morphometry and hip fracture (HF) patterns, and to assess the predictive value of radiographic morphometric parameters derived from standard anteroposterior radiographs.

**Materials and methods:**

A systematic review with meta-analysis was conducted in accordance with PRISMA guidelines. PubMed and MEDLINE databases were searched up to September 2025 for observational studies reporting radiographic morphometric parameters in patients with unilateral PFFs. Statistical meta-analysis was performed with random-effect models to compare intracapsular-ICF/subcapital-SCF fractures with intertrochanteric (ITF) and extracapsular (ECF) fracture patterns.

**Results:**

Twenty-two studies comprising 4184 patients (77.8% female; pooled mean age 77.4 years) were included. Meta-analysis using a random-effects model revealed that ITF were significantly associated with a smaller femoral head diameter (*p* < 0.001) and increased medial neck cortex thickness (*p* = 0.021) compared with ICF. ECF demonstrated significantly longer femoral neck axis length (*p* = 0.012), increased horizontal offset (*p* = 0.048), smaller absolute offset (*p* = 0.026) and shorter hip axis length (*p* = 0.026) than ICFs. Angular parameters provided the most robust stratification. ITFs and ECFs exhibited significantly lower (more varus) neck-shaft angles (*p* < 0.001) and significantly higher Wiberg angles (*p* < 0.001) compared with the ICF group.

**Conclusions:**

PF and HJ morphometry are associated with distinct HF patterns, independently of bone mineral density. Simple linear and angular measurements obtained from standard anteroposterior radiographs may assist fracture pattern stratification, risk assessment, and preoperative planning in elderly patients.

**Supplementary Information:**

The online version contains supplementary material available at 10.1007/s00276-026-03898-8.

## Introduction

The femur is the longest and strongest bone in the human body and plays a pivotal role in weight bearing and locomotion [21]. Anatomically, it comprises the proximal epiphysis, diaphysis, and distal epiphysis. The proximal femur (PF) encompasses the femoral head, neck, greater and lesser trochanters, and the intertrochanteric region, all of which are essential for the stability and function of the hip joint (HJ) [14]. The femoral head is nearly spherical and articulates with the acetabulum, while the femoral neck forms the neck shaft angle (NSA) and femoral anteversion angle (FAVA), two parameters that critically influence hip biomechanics, joint reaction forces, and load transfer across the PF [38]. Key morphometric parameters, including femoral head diameter (FHD), femoral neck length (FNL), NSA, and FAVA, directly influence stress distribution, bending moments, and torsional forces exerted on the HJ and PF [13]. A precise morphometric evaluation of the PF and hip is therefore crucial for prosthetic design, restoration of native hip biomechanics in arthroplasty, and the assessment of population-specific anatomical variations that may affect fracture risk [25, 43].

Proximal femoral fractures (PFFs) represent one of the most frequent fragility fractures in the elderly population and are a major cause of morbidity, functional impairment, and mortality, posing a substantial socioeconomic and healthcare burden worldwide [2]. These fractures are broadly classified into intracapsular (ICFs) and extracapsular (ECFs) types based on their anatomical relationship to the HJ capsule [1, 45]. Intracapsular fractures (ICFs) include subcapital, transcervical, and basicervical femoral neck fractures and are associated with an increased risk of avascular necrosis due to potential compromise of the femoral head blood supply [20]. Extracapsular fractures (ECFs) comprise intertrochanteric and subtrochanteric fractures (ITFs and STFs), which are biomechanically distinct, exhibiting different stability profiles and fixation requirements [26]. Early surgical management, most commonly through internal fixation or arthroplasty, is generally recommended to restore mechanical stability, allow early mobilization, and improve functional outcomes and prognosis [2].

Accumulating evidence indicates that morphometric characteristics of the PF and HJ significantly influence both fracture susceptibility and fracture pattern [7, 12]. Increased femoral neck width (FNW) and longer hip axis length (HAL) have been associated with a higher incidence of FNFs, whereas shorter HAL and larger acetabular angles (AAs) appear to correlate more strongly with ITF patterns [7, 12, 42]. Alterations in femoral offset and increased NSA have been reported in both ITF and SCF compared with non-fractured populations, suggesting a shared geometric predisposition that modifies load distribution and stress concentration within the PF [7]. Recent DXA- and CT-based structural studies have further demonstrated that proximal femoral geometry improves fracture prediction beyond bone mineral density alone [3, 17, 48]. In addition, more recent radiographic cohort studies have reported that increased Wiberg angle and reduced NSA are associated with a higher likelihood of pertrochanteric (PTF) or ECF localization in geriatric patients [35, 49]. These findings support the concept that PF and HJ morphometry, independent of bone mineral density, play a critical role in fracture biomechanics and may contribute to patient-specific risk stratification and targeted prevention strategies [12].

The purpose of the present study was to systematically investigate the association between PF and HJ morphometry and hip fracture (HF) patterns. In addition, this study aimed to evaluate whether simple linear and angular morphometric measurements obtained from standard anteroposterior radiographs could help predict HF risk and fracture type in the elderly population.

## Materials and methods

This systematic review with meta-analysis aimed to evaluate the association between PF and HJ morphometry and femoral fracture risk and was conducted in accordance with the Preferred Reporting Items for Systematic Reviews and Meta-Analyses (PRISMA) guidelines [28]. The study protocol was prospectively registered in the PROSPERO international prospective register of systematic reviews (registration number: CRD420251113271).

### Search strategy and eligibility criteria

A comprehensive literature search of the PubMed and MEDLINE databases was conducted through September 2025 to identify studies investigating PF and HJ morphometry and its association with PFFs. The search strategy combined the following terms using Boolean operators (AND/OR): “proximal femur”, “proximal hip”, “hip”, “morphometry”, “geometry”, “anatomical parameters”, “morphometric parameters”, “radiographic anatomy”, “radiographic morphometry”, “geometrical traits”, “fracture”, “fracture pattern”, “fracture classification”, and “proximal femur fracture”. Reference lists of all included articles were manually screened to identify additional eligible studies. Studies were excluded after full-text assessment, and the corresponding reasons for exclusion were documented.

### Selection criteria

Eligible studies included observational study designs, specifically retrospective or prospective cohort studies, case-control studies, and cross-sectional investigations reporting clinical and radiological data on PF and HJ morphometry in patients with unilateral PFFs. Radiographic assessment was performed either on the contralateral, non-fractured hip at the time of injury or on the fractured hip based on radiographs obtained prior to injury for other clinical indications. Exclusion criteria comprised studies not published in English, studies employing imaging modalities other than plain radiographs, studies that did not specify fracture type, and investigations conducted on cadaveric specimens. Expert opinions, book chapters, and conference abstracts were also excluded.

### Quality assessment of included studies

The methodological quality of the included studies was independently assessed by two reviewers using the Newcastle–Ottawa Scale (NOS). The NOS evaluates study quality using a star-based system (range 0–9), with higher scores indicating superior methodological quality. Assessment domains included selection of study groups (4 items), comparability of groups (1 item), and ascertainment of outcomes (3 items). In the comparability domain, up to 2 stars were awarded when studies reported patient demographics and provided an adequate radiological assessment of the hip, defined as an evaluation of at least 3 morphometric parameters. Discrepancies between reviewers were resolved through re-examination of the original articles and consensus discussion (Table 1).

**Table 1:** Methodological quality assessment of included studies using the Newcastle–Ottawa Scale (NOS)

Study (First author, country)	Year	Case definition	Representativeness	Selection of controls	Definition of controls	Comparability*	Ascertainment of exposure	Non-response rate	Total *
Ferris et al. [10], UK	1989	–	*	*	–	**	*	*	7
Gluer et al. [11], US	1994	–	*	*	–	*	*	*	6
Yang et al. [48], Taiwan	1999	–	*	*	–	**	*	*	7
Partanen et al. [30], Finland	2001	–	*	*	–	*	*	*	6
Pulkkinen et al. [33], Finland	2004	–	*	*	–	**	*	*	7
Patron et al. [31], UK	2006	–	*	*	–	**	*	*	7
Panula et al. [29], Finland	2008	–	*	*	–	*	*	*	6
Lektrakul and Ratarasan [22], Thailand	2009	–	*	*	–	**	*	-	6
Im and Lim [16], Korea	2010	–	*	*	–	**	*	*	7
Pulkkinen et al. [32], Finland	2010	–	*	*	–	*	*	-	5
Yamauchi et al. [46], Japan	2016	–	*	*	–	*	*	*	6
Kazemi et al. [19], Iran	2016	–	*	*	–	*	*	*	6
Tang et al. [40], Singapore	2017	–	*	*	–	**	*	*	7
Lima et al. [24], Brazil	2017	–	*	*	–	*	*	*	6
Tokyay et al. [42], Turkey	2017	–	*	*	–	*	*	*	6
Hu et al. [15], China	2018	–	*	*	–	**	*	*	7
Rotem et al. [34], Israael	2019	–	*	*	–	**	*	*	7
Thalmann et al. [41], Germany	2021	–	*	*	–	*	*	*	6
Senra et al. [39], Portugal	2022	–	*	*	–	**	*	*	7
Barrido and Bengzon [5], Philippines	2022	–	*	*	–	**	*	*	7
Vlachos et al. [44], Greece	2023	–	*	*	–	**	*	*	7
Cukurlu et al. [7], Turkey	2023	–	*	*	–	**	*	*	7

### Data extraction and synthesis

Study selection and data extraction were performed independently by the same two reviewers. Titles and abstracts were initially screened for eligibility, followed by full-text evaluation of potentially relevant studies. Extracted data included year of publication, country of origin, sample size, demographic characteristics (age and sex), and fracture type. PFFs were categorized into three subgroups: *subgroup 1*, subcapital or intracapsular fractures (SCFs OR ICFs); *subgroup 2*, intertrochanteric fractures (ITFs); and *subgroup 3*, extracapsular fractures (ECFs), including intertrochanteric and subtrochanteric fractures (ITF and STFs).

**Fig. 1:**
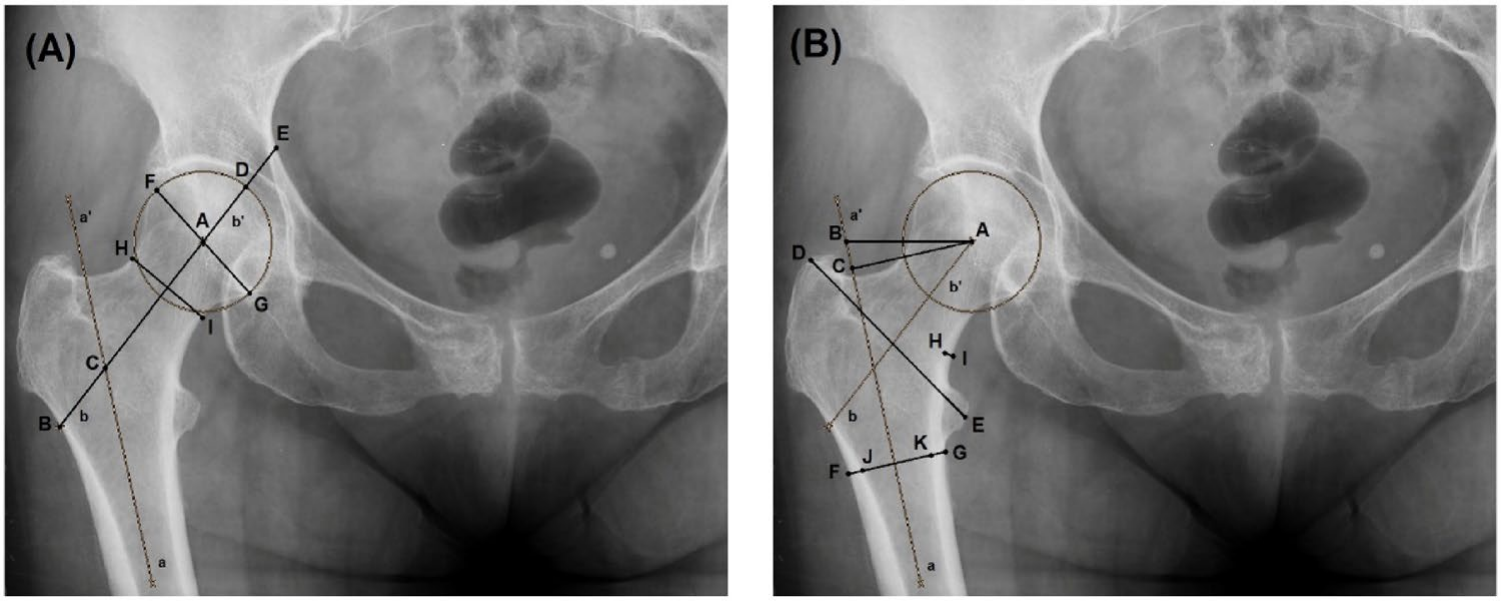
Anteroposterior X-Ray of the hip joint, Linear Parameters. **A **Femoral neck axis length (AB) is defined as the distance from the lateral femoral cortex (B) to the center of the femoral head (A), measured along the femoral neck axis and passing through the center of the femoral neck. Femoral neck length (AC) represents the distance from the anatomical axis of the femoral shaft (C) to the center of the femoral head (A), passing through the center of the femoral neck. Head–trochanter length (BD) is defined as the distance from the lateral femoral cortex (B) to the lateral margin of the femoral head (D), measured through the center of the femoral neck. Hip axis length (BE) is measured as the distance from the lateral femoral cortex (B) to the innermost margin of the pelvic rim (E), passing through the center of the femoral neck. Femoral head diameter (FG) is illustrated. Femoral neck width (HI) is defined as the shortest distance between the superolateral margin (H) and the inferomedial margin (I) of the femoral neck. **B** Horizontal offset (AB) is defined as the horizontal distance from the center of the femoral head (A) to the femoral shaft axis (b). Absolute offset (AC) represents the perpendicular distance from the center of the femoral head (A) to the femoral shaft axis (a). Intertrochanteric distance (DE) is measured as the distance from the tip of the greater trochanter (D) to the center of the lesser trochanter (E). Femoral shaft diameter (FG) is defined as the diameter of the femoral shaft just below the level of the lesser trochanter. Medial neck cortex thickness (HI) is measured as the distance between the lateral (H) and medial (I) margins of the inferomedial femoral neck cortex above the lesser trochanter. Femoral canal diameter (JK) is defined as the diameter of the femoral canal just below the lesser trochanter. The femoral shaft axis (a–a′) and femoral neck axis (b–b′) are indicated

**Fig. 2:**
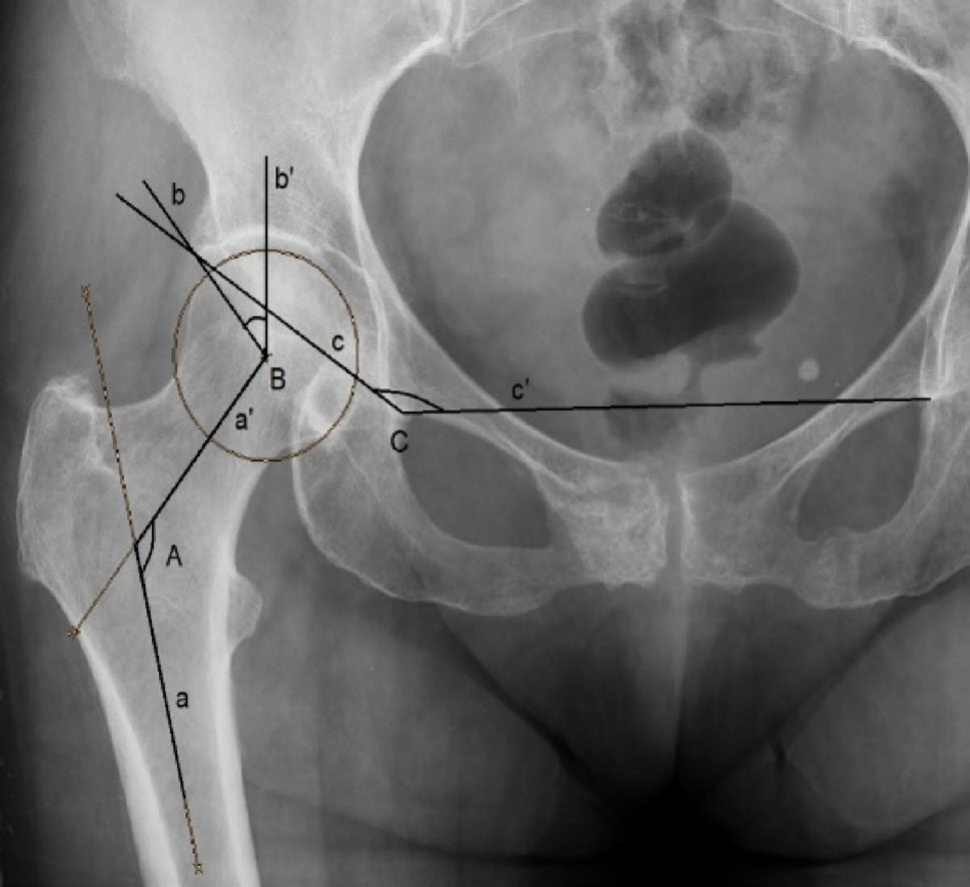
Anteroposterior radiograph of the hip joint illustrating angular morphometric parameters. The **neck–shaft angle (aAa′)** is defined as the angle between the femoral shaft axis (a) and the femoral neck axis (a′). The **Wiberg angle (bBb′)** is defined as the angle between the line connecting the center of the femoral head to the lateral margin of the acetabular roof (b) and a vertical reference line passing through the center of the femoral head along the longitudinal axis of the body (b′). The **acetabular angle (cCc′)** is defined as the angle between a line extending from the inferior margin of the unilateral teardrop through the most lateral margin of the acetabular roof (c) and a horizontal reference line connecting the inferior margins of the bilateral teardrops (c′). *a*: femoral shaft axis; *a′*: femoral neck axis

The evaluated morphometric parameters comprised: (1) femoral head diameter (FHD), (2) femoral neck length (FNL), (3) femoral neck width (FNW), (4) absolute offset (AO), (5) horizontal offset (HO), (6) intertrochanteric distance (ITD), (7) femoral diameter (FD) below the lesser trochanter, (8) femoral neck axis length (FNAL), (9) head trochanter length (HTL), (10) hip axis length (HAL), (11) femoral canal diameter (FCD) below the lesser trochanter, (12) medial neck cortex thickness (MNCT), (13) neck shaft angle (NSA), (14) Wiberg angle (WA), and (15) acetabular angle (AA). Definitions and measurement techniques for linear and angular parameters are illustrated in Figs. 1 and 2.

### Statistical analysis

Statistical analysis was performed using R software (R Core Team, 2014) and Python statistical libraries (Pandas, SciPy, and StatsModels). Formal meta-analysis was conducted for each morphometric parameter.

Proximal femoral and hip joint parameters were compared across three groups: subgroup 1 (intracapsular/subcapital fractures- ICFs), subgroup 2 (intertrochanteric fractures- ITFs), and subgroup 3 (extracapsular fractures- ECFs). For each parameter, the Mean Difference (MD) and its associated 95% Confidence Interval (CI) were calculated using a Random-Effects Model (DerSimonian-Laird method). Study precision was determined by the inverse of the variance, ensuring that larger, more robust studies contributed appropriately to the pooled estimate. Inter-study heterogeneity was quantitatively assessed using the Cochran’s Q statistic and the I^2^ index. Heterogeneity was categorized as low (I^2^ < 25%), moderate (25%–75%), or high (I^2^ >75%). To assess the risk of publication bias and small-study effects, funnel Plots were generated for all parameters with at least 10 study comparisons. Visual asymmetry was statistically verified using the Thompson and Sharp regression test [37]. Statistical significance for all tests was defined as a two-sided p-value < 0.05. For parameters where only a single study was available, results were reported descriptively or using fixed-effect models where appropriate.

## Results

**Fig. 3:**
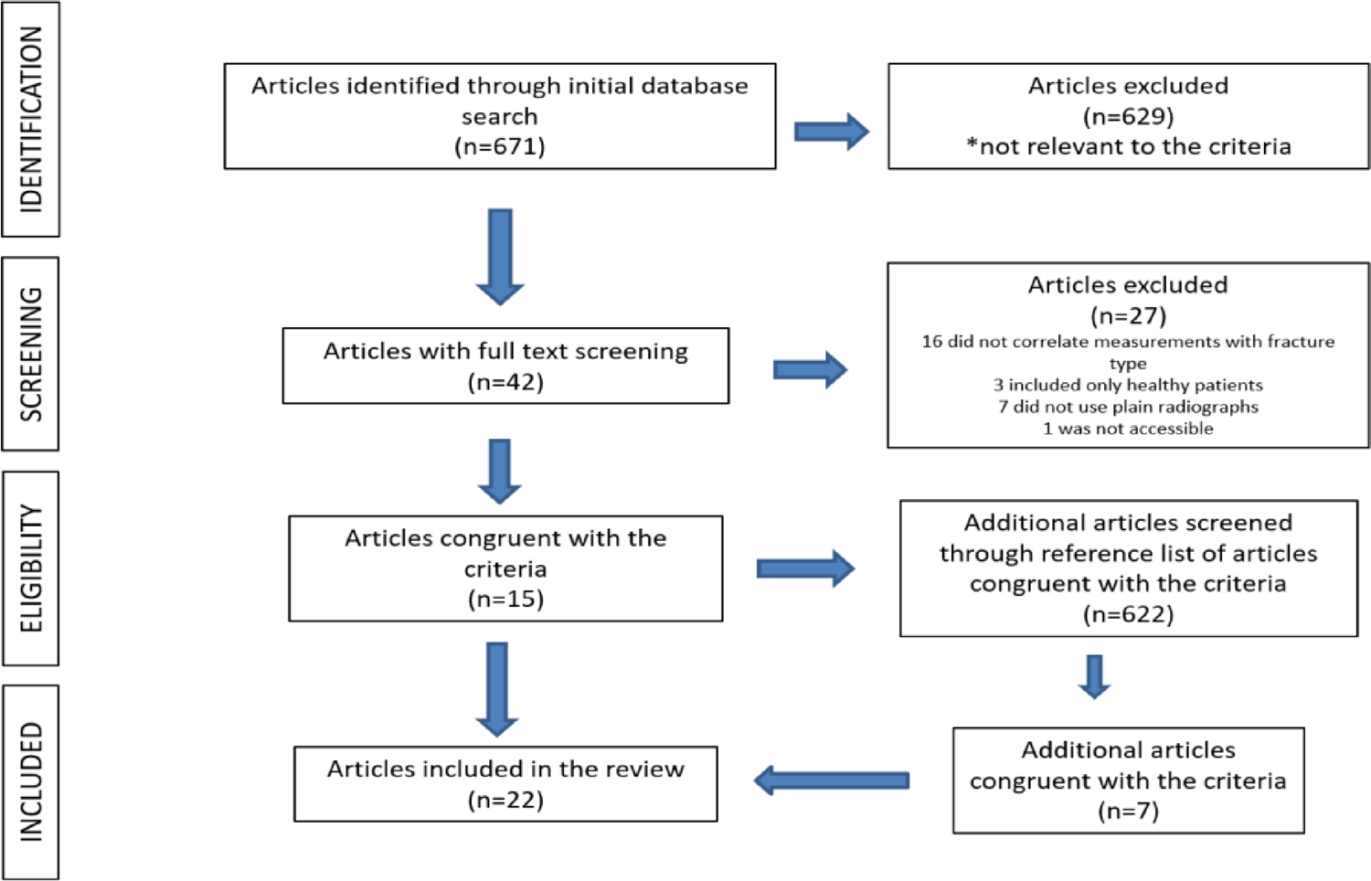
Flow diagram illustrating study identification, screening, eligibility assessment, and final inclusion of studies in accordance with the PRISMA guidelines. A total of 671 records were identified through database searching, with 22 studies ultimately included in the systematic review and pooled analysis

A total of 671 studies were identified through the initial database search. After removal of duplicates, titles, and abstracts were screened, 629 articles were excluded for not meeting the eligibility criteria. Following full-text assessment, 15 studies fulfilled the inclusion criteria [5, 7, 15, 16, 19, 22, 24, 29, 30, 32, 33, 39, 41, 44, 47]. During this stage, 27 studies were excluded: in 16 studies, fracture type was not correlated with radiographic morphometric parameters; in three studies, measurements were performed exclusively in healthy populations; in seven studies, imaging modalities other than plain radiographs were used; and one study was not accessible. An additional seven eligible studies were identified through manual screening of reference lists [10, 11, 31, 34, 40, 42, 46]. Ultimately, 22 studies were included in the final analysis [5, 7, 10, 11, 15, 16, 19, 22, 24, 29–34, 39–42, 44, 46, 47]. The study selection process is summarized in Fig. 3.

### Patient characteristics

All included studies reported the number of patients, sex distribution, and mean age. Overall, 4184 patients were analyzed, comprising 927 males (22.2%) and 3257 females (77.8%). Eleven studies reported mean age data for 1481 patients, regardless of fracture type, yielding a pooled mean age of 77.4 years.

Subgroup-specific analyses showed that 20 studies reported data for 2518 patients with SCFs (ICFs) of the femur, with a pooled mean age of 77.3 ± 9.0 years. Sixteen studies included 1469 patients with ITFs, with a pooled mean age of 80.3 ± 8.1 years. Eighteen studies reported data on 1513 patients with ECFs, with a pooled mean age of 80.3 ± 8.1 years.

### Linear parameters (Table 2)

**Table 2:** Summary of associations between linear morphometric parameters and hip fracture patterns.

Parameter	Comparison	Studies	MD	95% CI	*P*-value	I^2^ (%)
FHD	IC vs. IT	20	–0.58	[–0.86, –0.30]	< 0.001*	1.51
	IC vs. EC	22	–0.38	[–0.77, 0.01]	0.054	38.74
FNL	IC vs. IT	3	0.96	[–0.52, 2.44]	0.202	27.51
FNW	IC vs. IT	20	0.36	[–0.17, 0.89]	0.183	79.45
	IC vs. EC	26	0.51	[–0.06, 1.09]	0.081	94.77
AO	IC vs. IT	10	–0.77	[–1.91, 0.37]	0.186	73.74
	IC vs. EC	12	–0.92	[–1.73, –0.11]	0.026*	70.31
HO	IC vs. IT	2	0.20	[–1.27, 1.67]	0.789	0.0
	IC vs. EC	4	1.71	[0.02, 3.41]	0.048*	63.61
FD	IC vs. IT	3	0.01	[–0.18, 0.19]	0.923	58.15
FNAL	IC vs. IT	6	0.76	[–0.02, 1.55]	0.057	0.0
	IC vs. EC	8	1.51	[0.33, 2.70]	0.012*	60.89
HTL	IC vs. IT	7	0.32	[–1.47, 2.11]	0.726	64.84
HAL	IC vs. IT	14	–0.70	[–2.20, 0.80]	0.360	74.95
	IC vs. EC	16	–1.24	[–2.34, –0.15]	0.026*	84.22
FCD	IC vs. IT	1	0.10	[–0.04, 0.24]	0.167	0.0
MNCT	IC vs. IT	3	0.29	[0.04, 0.53]	0.021*	0.0

*HTL: head–trochanter length; IΤD: intertrochanteric distance; FD: femoral diameter below the lesser trochanter; FNAL: femoral neck axis length; FCD: femoral canal diameter below the lesser trochanter

** ID: Intertrochanteric distance, FD: Femoral diameter below the lesser trochanter, FCD: Femoral canal diameter below the lesser trochanter

IC: intracapsular/subcapital, IT: intertrochanteric, EC: extracapsularStatistically significant results are presented with asterisk (*)

#### Comparison between subgroup 1 (intracapsular/subcapital) and subgroup 2 (intertrochanteric)

**Fig. 4:**
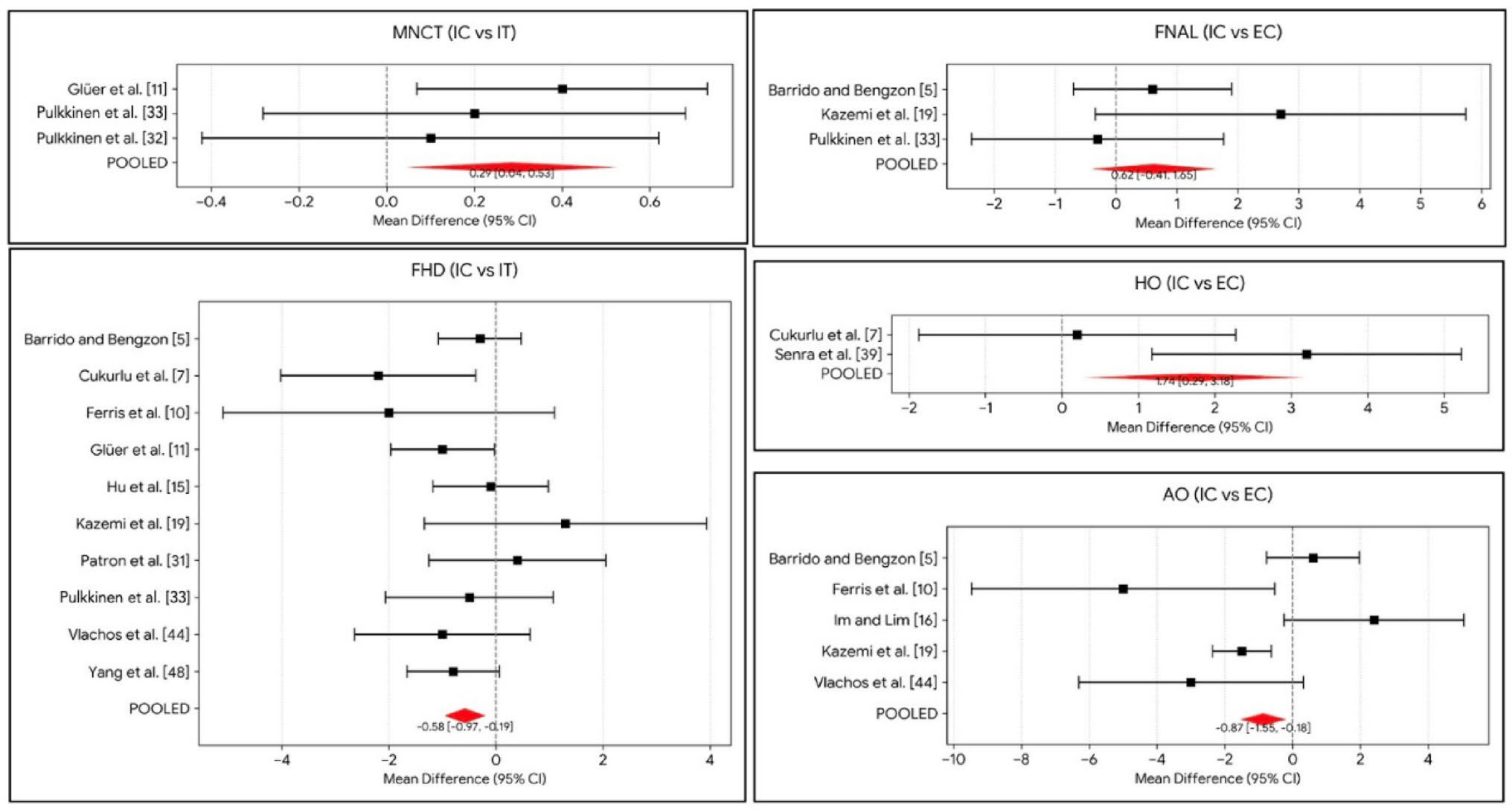
Forrest plots for the statistically significant results of the linear parameters

Medial neck cortex thickness (MNCT) was evaluated in 289 fracture cases. ITFs demonstrated significantly increased medial neck cortex thickness (MNCT) [MD = 0.29 mm (0.04–0.53); *p* = 0.021] (Fig. 4).

ITFs were associated with a significantly smaller FHD compared to ICFs [MD = –0.58 mm (–0.86 to –0.30); *p* < 0.001] with low heterogeneity (I^2^ = 1.51%) (Fig. 4).

No significant differences were observed for FNL, FNW, AO, HO, or HAL in this specific comparison (Table 2).

#### Comparison between subgroup 1 (intracapsular/subcapital) and subgroup 3 (extracapsular)

FNAL was evaluated in 282 patients. ECFs were characterized by significantly longer FNAL [MD = 1.51 mm (0.33 to 2.70); *p* = 0.012] (Fig. 4).

Horizontal offset (HO), assessed in 1149 patients. ECFs were characterized by significantly higher HO [MD = 1.71 mm (0.02–3.41); *p* = 0.048] (Fig. 4).

AO was analyzed in 781 cases. ECFs were associated with significantly smaller AO [MD = –0.92 mm (–1.73 to –0.11); *p* = 0.026] and shorter hip axis length (HAL) [MD = –1.24 mm (–2.34 to –0.15); *p* = 0.026] compared to ICFs (Fig. 4).

No statistically significant correlations were identified for FNW, or FHD (Table 2).

Funnel plot tests for possible publication bias were not statistically significant for all the parameters, except from FNW and HAL that suggested possible bias (Supplementary Materials).

#### Angular parameters

Angular parameters showed the most consistent associations with fracture pattern stratification across the included studies (Table 3).

**Table 3:** Summary of associations between angular morphometric parameters and hip fracture patterns. Statistically significant results are presented with asterisk (*)

Parameter	Comparisons	k	Pooled MD	95% CI Lower	95% CI Upper	*p*-value	I2 (%)
NSA	IC vs. IT	15	–2.05	–3.55	–0.55	0.007*	90.4
	IC vs. EC	17	–2.14	–3.20	–1.07	< 0.001*	90.3
WA	IC vs. IT	4	3.71	1.99	5.43	< 0.001*	53.8
	IC vs. EC	5	2.85	0.43	5.28	0.0210*	91.2
AA	IC vs. IT	2	2.80	–2.30	7.90	0.2815	93.7

*IC/SC: intracapsular/subcapital, IT: intertrochanteric, EC: extracapsular

** NSA: Neck-Shaft Angle; WA: Wiberg Angle; HAL: Hip Axis Length; AA: Acetabular Angle

**Fig. 5:**
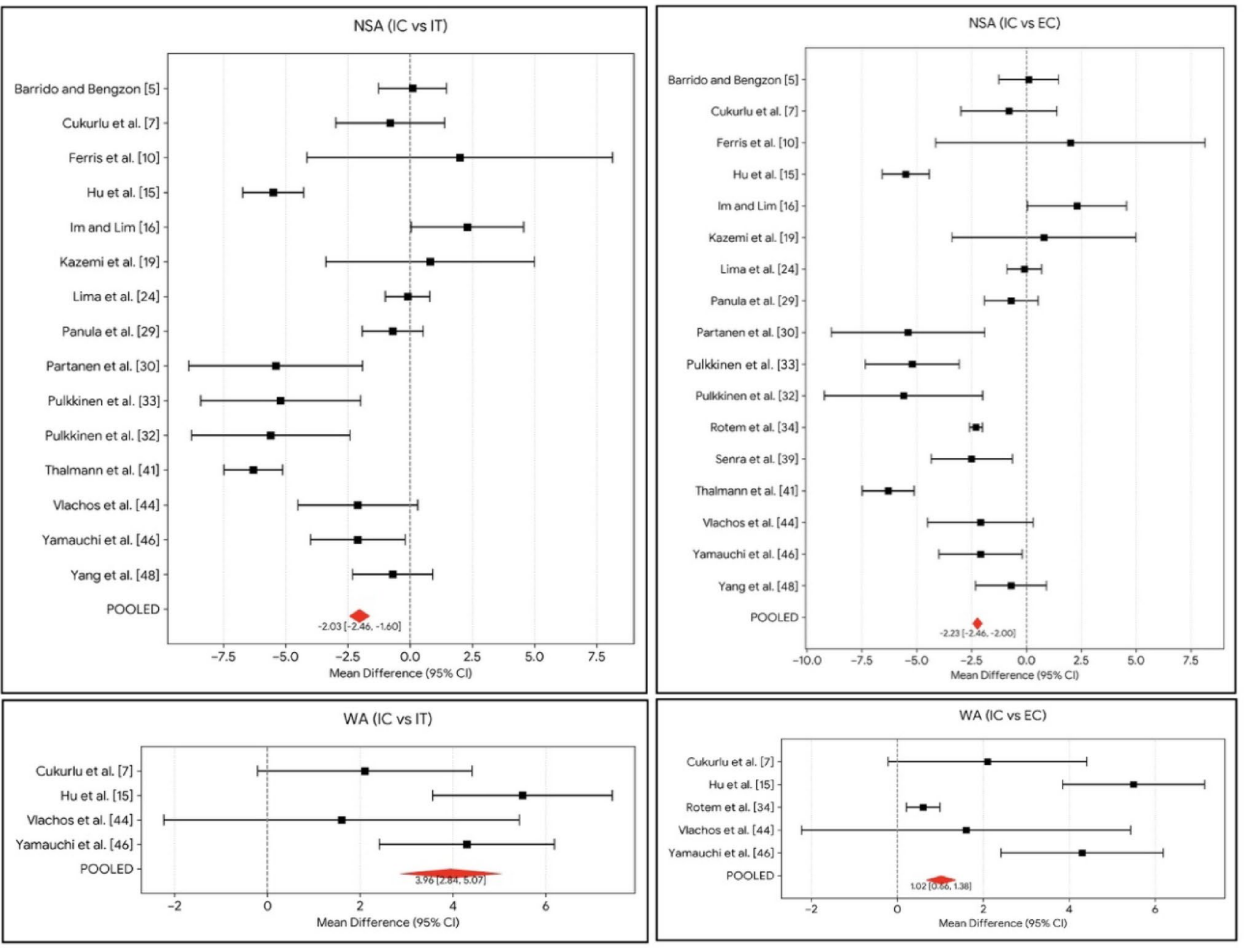
Forrest plots for the statistically significant results of the angular parameters

A more varus orientation of the femoral neck was a robust predictor of ECFs and ITFs patterns. NSA was significantly lower in the ITF group [MD = −2.05° (−3.55 to −0.55); *p* = 0.007] and the ECF group [MD = −2.14° (−3.20 to −1.07); *p* < 0.001] compared to the ICF group (Fig. 5).

Patients with ITF demonstrated a significantly higher Wiberg angle (WA) compared to ICF [MD = 3.71° (1.99 to 5.43); *p* < 0.001]. Similarly, the ECF group showed higher WA values than the ICF group [MD = 2.85° (0.43 to 5.28); *p* = 0.021] (Fig. 5).

No statistically significant differences were found for the acetabular angle (AA) between ICF and ITF groups (Table 3).

Funnel plot tests for possible publication bias were not statistically significant for all the parameters (Supplementary Materials).

## Discussion

Proximal femur and HJ morphometry have been consistently associated with distinct HF patterns [7, 12]. The present study builds on a systematic review with meta-analysis demonstrating that PF and HJ morphometry are significantly associated with distinct HF patterns, independently of bone mineral density [17, 48]. Specific linear and angular geometric parameters influence load distribution and fracture susceptibility and can be reliably assessed using standard anteroposterior radiographs. Recent DXA- and CT-based structural studies further support that geometric assessment improves fracture prediction beyond densitometric evaluation alone [3, 27]. By synthesizing high-quality evidence and focusing on simple, reproducible radiographic measurements, this work addresses an important and underexplored determinant of HF heterogeneity. The findings support improved fracture risk stratification, prognostic assessment, and preoperative planning, including implant templating and offset restoration [4, 6], thereby enhancing fracture prevention strategies and optimizing surgical decision-making in the elderly population.

Based on the meta-analysis, ITFs were associated with increased MNCT, smaller FHD, lower NSA, and higher WA compared with ICFs. ECFs demonstrated significantly longer FNAL, increased HO, smaller AO, and shorter HAL relative to ICFs, together with lower NSA and higher WA. These morphometric configurations may predispose the proximal femur to increased extracapsular loading. In contrast, ICFs were characterized by relatively longer HAL and larger FHD. Notably, lower NSA consistently emerged as a recurrent predictor of extracapsular fracture patterns across multiple studies [7, 12, 15, 16, 19, 24, 29, 30, 34, 39]. More recent cohort studies have similarly shown that increased WA and reduced NSA are associated with a higher likelihood of pertrochanteric or extracapsular fracture localization in geriatric populations [35, 49]. Collectively, these findings indicate that PF and HJ morphometry play a substantial role in fracture susceptibility, independently of bone mineral density.

Evaluation of PF and HJ morphometry is currently performed using plain radiographs, computed tomography scan (CT), and dual-energy X-ray absorptiometry (DXA). CT allows detailed three-dimensional (3D) assessment, while DXA enables combined evaluation of bone mineral density and geometric parameters relevant to fracture risk prediction [12, 32, 33]. Recent DXA-based structural engineering, hip structural analysis, and finite-element studies have further shown that geometric information embedded in routine DXA scans improves the discrimination of patients at risk of hip fracture beyond that provided by BMD alone [27, 48]. Similarly, CT-based investigations demonstrated that three-dimensional proximal femoral geometry, cortical distribution, and density mapping are independently associated with hip fracture susceptibility [3, 17]. However, most comparative studies investigating morphometric differences between HF subtypes rely on standard anteroposterior radiographs, which remain the most practical modality in acute clinical settings [7, 15, 24, 30, 31]. Plain radiographs are widely available, cost-effective, and associated with minimal radiation exposure, allowing rapid assessment of key morphometric parameters [31]. Their accessibility supports routine prognostic evaluation, facilitates early stratification of fracture patterns, and enables integration of morphometric assessment into everyday clinical practice [18].

The present meta-analysis demonstrated that ECFs of the femur exhibit significantly longer FNAL and increased HO, together with smaller AO and shorter HAL compared with ICFs. In direct comparisons between ICFs and ITFs, ITFs were characterized solely by increased MNCT. From a biomechanical perspective, increased MNCT may enhance local stiffness and redirect load transmission toward the intertrochanteric region [12]. In ECFs, longer FNAL combined with reduced AO may amplify bending moments and varus stresses across the trochanteric region, whereas a shorter HAL may further concentrate load away from the femoral head neck junction [7, 12, 29, 30, 42]. Collectively, these morphometric configurations provide a plausible biomechanical basis for the preferential development of extracapsular fracture patterns.

Femoral offset (FO) evaluation has important clinical implications, as inadequate restoration has been associated with altered hip biomechanics and postoperative complications, including abductor muscle dysfunction and trochanteric pain syndromes [9]. Recent arthroplasty studies have also confirmed that a mismatch between planned and restored offset may negatively affect postoperative functional outcomes, gait symmetry, and hip stability [4, 36]. On standard anteroposterior radiographs, HO represents a projected horizontal measurement and is sensitive to femoral rotation, abduction, and adduction, which may introduce clinically relevant error [8, 23]. In contrast, AO, defined as the perpendicular distance from the hip center of rotation to the longitudinal axis of the femoral shaft, represents the anatomical reference for FO and is affected by femoral rotation but not by abduction or adduction [23]. Notably, reduced AO in ECFs suggests diminished true femoral offset, which may increase abductor loading and bending stresses across the trochanteric region, thereby contributing to extracapsular fracture patterns. Consequently, AO appears to have greater clinical relevance and surgical importance, owing to its reduced susceptibility to positional variation and its more accurate representation of femoral geometry on plain radiographs.

In hip reconstructive procedures, including total hip arthroplasty and hip hemiarthroplasty, preoperative assessment of AO on standard anteroposterior radiographs may assist implant selection and reduce the risk of postoperative complications related to HJ lateralization, such as abductor dysfunction and trochanteric pain syndromes [8, 23]. Contemporary three-dimensional templating studies suggest that CT-based planning may further improve implant sizing and offset restoration compared with conventional two-dimensional methods [6]. Measurement of the perpendicular distance between the hip center of rotation and the femoral shaft axis can be reliably reproduced intraoperatively, facilitating restoration of native hip biomechanics [8, 23]. Accordingly, absolute offset represents a surgically relevant parameter for implant selection and biomechanical restoration.

Furthermore, the present analysis demonstrated that ECFs were associated with higher Wiberg angles and lower neck–shaft angles compared with ICFs. Similar angular trends were observed in ITFs. These findings are consistent with radiographic series reporting relatively varus neck orientation and increased Wiberg angles in trochanteric fractures compared with cervical fractures [12, 29, 30, 39, 42, 44]. Biomechanically, these angular configurations increase superolateral femoral head coverage and shift joint reaction forces laterally, promoting shear and bending stresses in the trochanteric region [7, 12, 29, 30, 42]. Routine measurement of NSA and WA on standard anteroposterior radiographs may therefore enhance fracture risk stratification and facilitate anticipation of fracture subtype in elderly patients [12, 29, 30, 32, 42]. Incorporation of angular morphometry into preoperative planning may also assist implant selection and alignment, optimize restoration of hip anatomy and biomechanics, and potentially improve clinical outcomes [7, 12, 30, 34, 39, 42].

This study has several limitations. First, exclusive reliance on standard anteroposterior radiographs limited the analysis to two-dimensional measurements, whereas CT and dual-energy X-ray absorptiometry (DXA) may provide more comprehensive three-dimensional anatomical assessment, along with bone-quality information. Second, variation in patient positioning and femoral rotation during radiographic acquisition may have introduced measurement variability across studies. Third, substantial statistical heterogeneity was observed for several key parameters, particularly the angular measurements of NSA and WA, likely reflecting differences in study populations, geographic regions, radiographic protocols, and measurement techniques. Fourth, several pooled analyses were based on a limited number of studies (k = 1–3), particularly for parameters such as MNCT, FNAL, FCD, HO, and AA. These estimates should therefore be interpreted cautiously, as small-study meta-analyses are more susceptible to imprecision, unstable effect sizes, and limited assessment of publication bias. Finally, although the overall pooled population was substantial, most included studies originated from European and Asian cohorts. Accordingly, the findings may not be fully generalizable to other ethnic populations with distinct proximal femoral anatomical characteristics.

Despite these limitations, this systematic review has notable strengths. It provides a comprehensive synthesis of available evidence examining the relationship between PF and HJ morphometry and HF patterns using clearly defined radiographic parameters. Rigorous methodology was applied, including adherence to PRISMA guidelines, independent study selection and quality assessment, and systematic data extraction. By focusing on comparable measurements derived from plain radiographs, the findings offer clinically applicable insights for everyday orthopedic practice. Moreover, this represents one of the largest systematic reviews with meta-analysis on this topic to date, incorporating a substantial pooled population, thereby enhancing the robustness of the conclusions. In routine orthopedic practice, proximal femur morphometry can be rapidly obtained from admission radiographs at no additional cost or imaging burden. Integration of these measurements into geriatric fracture pathways may help identify patients at risk of unstable ECFs, anticipate fixation complexity, and optimize implant inventory selection. From a clinical perspective, recognition of patient-specific proximal femoral geometry may improve identification of elderly individuals at increased risk for specific hip fracture patterns. Incorporation of simple radiographic morphometric parameters into routine assessment may support individualized prevention strategies, optimize implant selection, and enhance surgical planning. Future prospective studies integrating morphometry with bone mineral density, frailty indices, and fall-risk variables may further improve predictive models for hip fracture prevention.

## Conclusion

Proximal femoral and hip morphometry are associated with specific HF patterns. Lower NSA and higher WA were the most consistent predictors of ECF types. Simple radiographic geometric measurements may complement clinical assessment for fracture-risk stratification, surgical planning, and implant selection. Further prospective multicenter studies are needed to validate these findings.

## Supplementary Information

Below is the link to the electronic supplementary material.

Supplementary Material 1

## Data Availability

Please contact the authors for data requests.
