# Supplementary material for: Proximal femoral morphometry and its association with hip fracture patterns: a systematic review with meta-analysis
Source: Surg Radiol Anat. 2026 May 22;48(1):129. doi: 10.1007/s00276-026-03898-8 (PMC13186906; doi:10.1007/s00276-026-03898-8)
Supplement: Supplementary file 1 — Supplementary Material 1 [file 276_2026_3898_MOESM1_ESM.docx]

**Supplementary Materials**


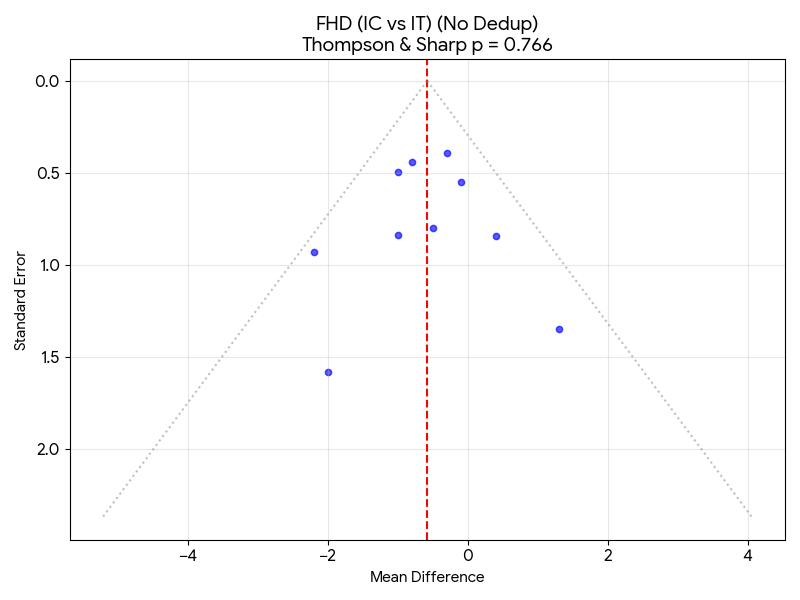


Supplementary Figure 1. Funnel plot with Thompson & Sharp test for asymmetry for the femoral head diameter (FHD) comparison between the intracapsular (IC) versus intetrochanteric (IT). No publication bias detected (p=0.766).


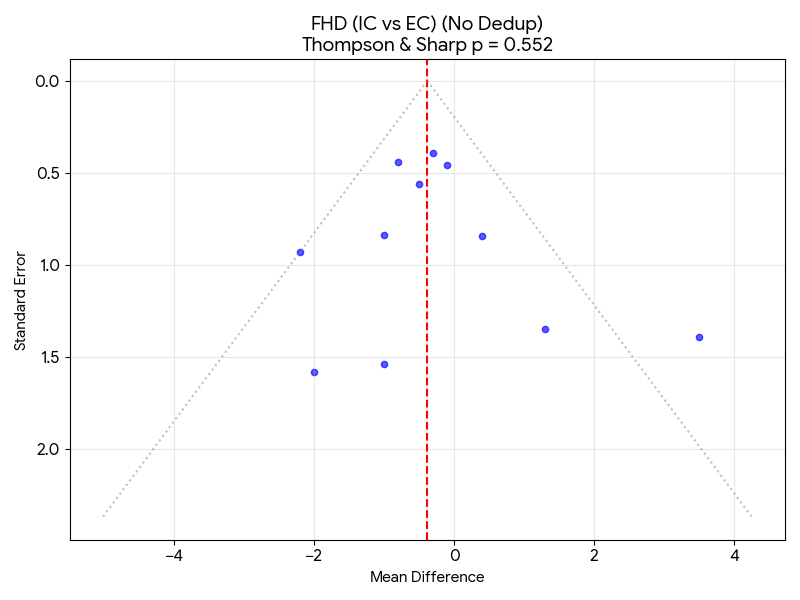


Supplementary Figure 2. Funnel plot with Thompson & Sharp test for asymmetry for the femoral head diameter (FHD) comparison between the intracapsular (IC) versus extracapsular (EC). No publication bias detected (p=0.552).


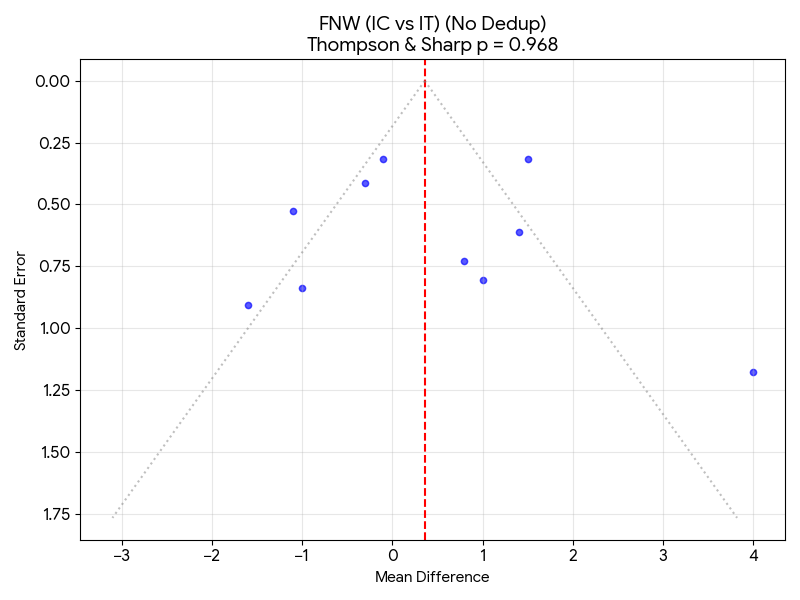


Supplementary Figure 3. Funnel plot with Thompson & Sharp test for asymmetry for the femoral neck width (FNW). comparison between the intracapsular (IC) versus intetrochanteric (IT). No publication bias detected (p=0.968).


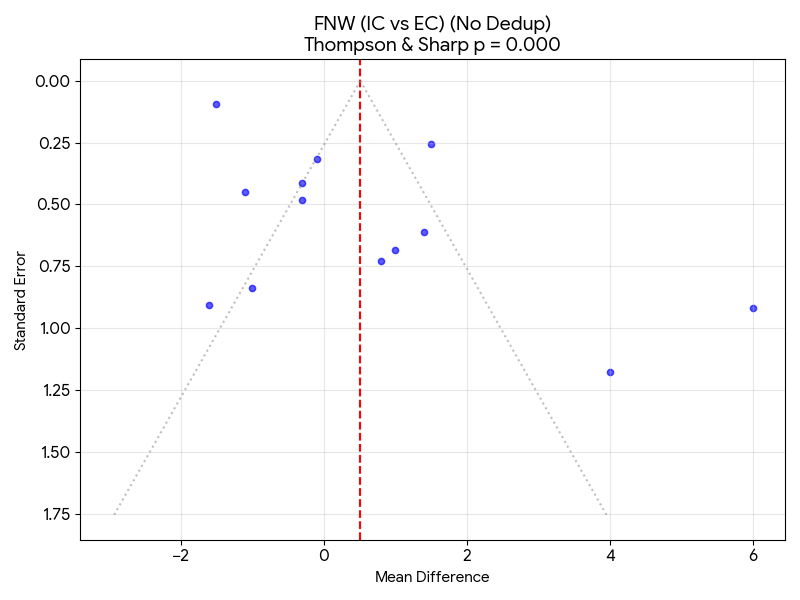


Supplementary Figure 4. Funnel plot with Thompson & Sharp test for asymmetry for the femoral neck width (FNW) comparison between the intracapsular (IC) versus extracapsular (EC). Possible publication bias detected (p<0.001).


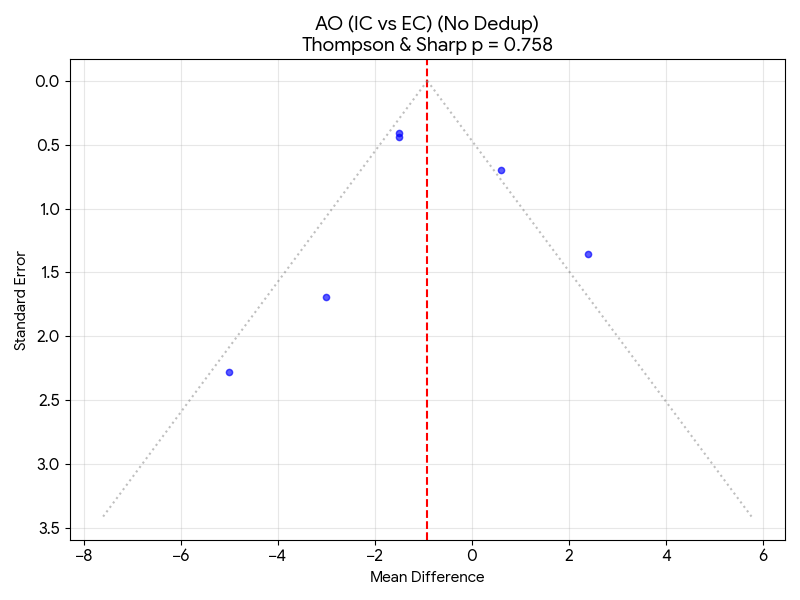


Supplementary Figure 5. Funnel plot with Thompson & Sharp test for asymmetry for the absolute offset (AO) comparison between the intracapsular (IC) versus extracapsular (EC). No publication bias detected (p=0.758).


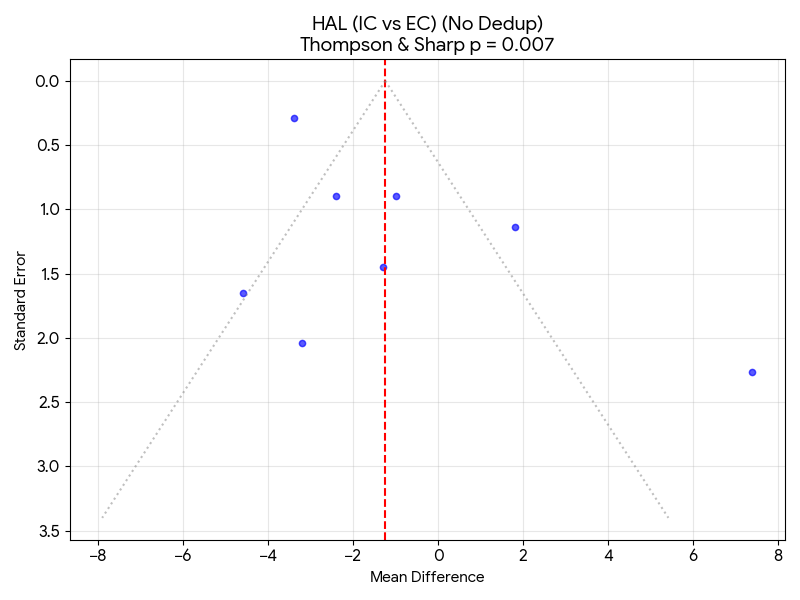


Supplementary Figure 6. Funnel plot with Thompson & Sharp test for asymmetry for the hip axis length (HAL) comparison between the intracapsular (IC) versus extracapsular (EC). Possible publication bias detected (p=0.007).


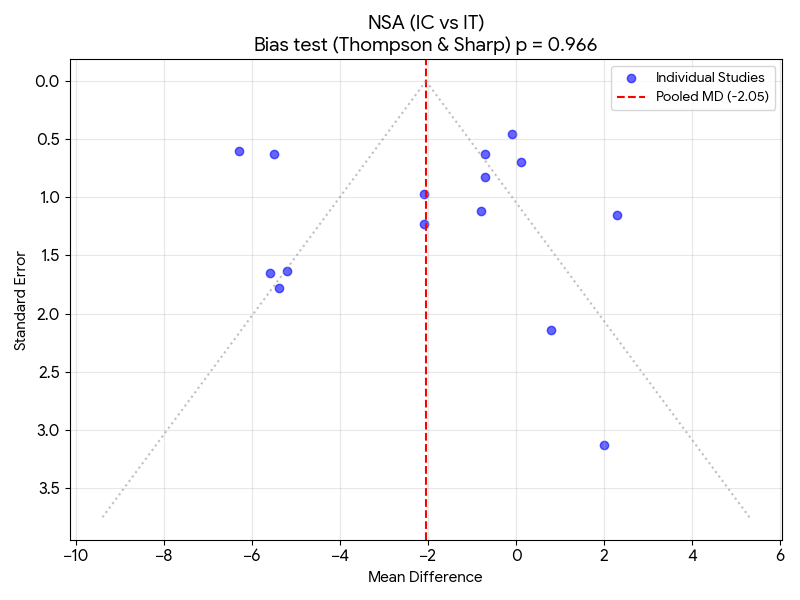


Supplementary Figure 7. Funnel plot with Thompson & Sharp test for asymmetry for the Neck-Shaft Angle (NSA) comparison between the intracapsular (IC) versus intetrochanteric (IT). No publication bias detected (p=0.966).


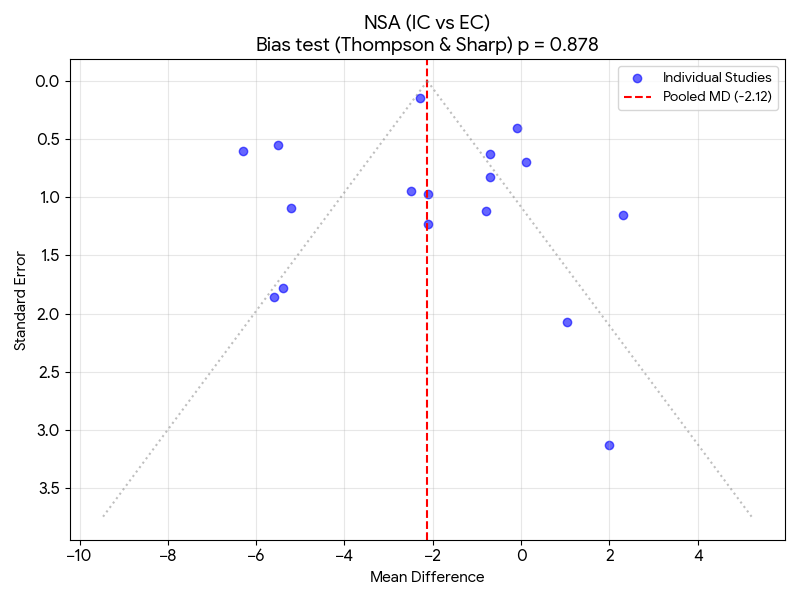


Supplementary Figure 8. Funnel plot with Thompson & Sharp test for asymmetry for the Neck-Shaft Angle (NSA) comparison between the intracapsular (IC) versus extracapsular (EC). No publication bias detected (p=0.878).
